# Editorial: Empirical Research at a Distance: New Methods for Developmental Science

**DOI:** 10.3389/fpsyg.2022.938995

**Published:** 2022-05-25

**Authors:** Sho Tsuji, Dima Amso, Rhodri Cusack, Natasha Kirkham, Lisa M. Oakes

**Affiliations:** ^1^International Research Center for Neurointelligence, The University of Tokyo Institutes for Advanced Study, The University of Tokyo, Tokyo, Japan; ^2^Institute for AI and Beyond, The University of Tokyo, Tokyo, Japan; ^3^Developmental Cognitive Neuroscience Laboratory, Department of Psychology, Columbia University, New York, NY, United States; ^4^Trinity College Institute of Neuroscience, Trinity College Dublin, Dublin, Ireland; ^5^Centre for Brain and Cognitive Development, Department of Psychological Sciences, Birkbeck, University of London, London, United Kingdom; ^6^Center for Mind and Brain, Department of Psychology, University of California, Davis, Davis, CA, United States

**Keywords:** online testing, developmental psychology, remote testing, new methods, child development, COVID-19

## Introduction

The COVID-19 pandemic presented many challenges for the research community. The collection of papers in this Research Topic illustrate how developmental scientists met those challenges and created clever and innovative methods to continue research when it was not safe to have children and families physically in the lab. Soon after labs were closed by universities and institutions, developmental scientists were scheduling video conferences with children to collect data, programming web-based procedures for participation, and considering ways to reevaluate previously collected data. The papers presented here demonstrate how the community continued to conduct research even though we were not able to work directly with our participants.

These papers reflect a diverse set of approaches to studying a wide range of content. They not only demonstrate the effectiveness (or ineffectiveness) of these methods, but also engage discussion on their drawbacks and gains. Are there advantages of new online paradigms with respect to increasing our reach to wider participant pools than usually recruited? If so, do these advantages outweigh the very real disadvantages of a decrease in the precision of measurements (e.g., not being able to control for distraction in the testing environment)? What criteria would our field need to develop for the adoption of such new methods (e.g., privacy concerns, ethical considerations)? Liu et al. discuss the benefits of reaching out into the community to find collaboration and to engage with participants regarding research ethics and values.

This Editorial is organized as follows. First, we describe the wide range of methods and measures adopted, illustrating how the move to collecting data at a distance did not restrict the ways we conducted research or the questions we asked. Next, we describe efforts to directly compare the results of data collected online (both supervised and unsupervised) to data collected in person. This Research Topic of papers reveals both findings that are context-independent (i.e., the same pattern is observed regardless of how the data were collected) and context-dependent (i.e., different patterns are observed in online vs. in-person data). In addition, these papers address questions of how procedures need to be modified, differences in data quality, and what measures can and cannot be assessed in different data collection contexts. We then present “lessons learned” and advice for best practices. We suspect that developmental scientists will continue to collect data at a distance, and the work presented here can provide guidelines to ensure that future efforts produce high quality work. Finally, we discuss what online remote research can offer–and what it cannot–as the field moves forward.

## The Range of Methods and Measures

Researchers who were unable to collect data in person adopted a number of different approaches to continue their research. Some explored ways of analyzing previously collected data. For example, Solby et al. applied neural network analyses to archival data on infants' problem solving abilities. Mendoza and Fausey provide guidance for manually annotating children's everyday experiences from data in repositories. Many others began developing or using tools for collecting data remotely. For some researchers this meant creating versions of their experimental procedures that could be administered in a moderated video conference (e.g., using a platform such as Zoom). Other researchers used or developed procedures for unsupervised data collection, in which the participants or families used their own computers or equipment provided by the researchers to collect data in their own homes (e.g., the online experimental platform Gorilla). We next describe the work conducted with moderated and unmoderated procedures.

### Moderated Procedures

Many of the papers in this collection provide examples of moderated or synchronous remote data collection. In these procedures, participants typically make an appointment and meet with the researcher remotely *via* a video conferencing platform. This is essential when the experimental paradigm requires that children interact with and respond to instructions given by a researcher. Researchers used this approach to investigate a wide range of questions, including school aged children's solutions to balance beam problems (Filion and Sirois), young children's performance on traditional false belief tasks (Schidelko et al.), mother-infant interaction (McElwain et al.), and standardized cognitive functioning assessments like Mullen or Bayleys (Krogh-Jesperson et al.).

Moderated sessions also can be less structured in order to capture more “naturalistic” behaviors at home. Moderated sessions have been used to record free-play with parents and infants (Shin et al.; Segal and Moulson), puzzle play with preschoolers and parents (Pochinki et al.), and eating behaviors at mealtime (Venkatesh and DeJesus). In a semi-structured approach, Woon et al. recorded parents reading a book with their infants or toddlers, using the screen sharing feature on Zoom to present the same book to all participants.

There are also examples of researchers conducting multi-session and training studies using fully remote experimenter-moderated sessions. Bambha and Casasola had an experimenter meet with children on Zoom, every week for 5 weeks, to deliver a spatio-cognitive and visuo-motor skill training protocol. Ozernov-Palchik et al. delivered a fully remote language intervention and assessed its impact. Both papers discuss the challenges and strengths of such a multi-session remote approach.

Because they allow for better monitoring of caregiver and child variables, some researchers chose moderated sessions for tasks that could have been conducted in unmoderated sessions, including looking-time procedures with young children (Bacon et al.; Chuey et al.; Morini and Blair) and monitoring children completing tasks using Qualtrics (Qualtrics, 2022) and other software (Segura and Pompéia; Vales et al.). Researchers choose moderated sessions for a variety of reasons including ease of setting up the procedure, a desire for more control, targeting a particular population, and comparing results between moderated and unmoderated studies.

### Unmoderated Procedures

Many researchers elected to conduct unmoderated or asynchronous remote data collection, especially for screen-based, non-interactive experimental tasks. Platforms such as Lookit (Scott and Schulz, 2017) facilitated the administration of infant looking time tasks, in which researchers can set up stimuli to present to infants or young children and record their looking to those stimuli. Platforms such as Gorilla (Anwyl-Irvine et al., 2020) or LabVanced (Finger et al., 2017) allow researchers to design and program experiments to collect reaction time and accuracy as children press keys on their computer keyboard in response to stimuli presented on the monitor. These unmoderated procedures have the advantage that participants can log into an experimental program over a web browser and participate in an experiment in their own time by following the screen prompts. Oftentimes, the experimental software allows tight control over experimental variables like stimulus presentation and timing. They have the disadvantage that there is no experimenter to direct the parent or child, to make sure that the setting and recording is optimal, and to ensure compliance with the task. Nevertheless, several papers in this Research Topic demonstrate that these can be effective procedures.

For example, Nelson and Oakes demonstrated that infants' visual preference can be examined using the unmoderated platform Lookit and labor-intensive off-line coding. Others presented procedures that code looking automatically, either online or after data recording. Using the built-in webcam-based automatic eye tracking feature of LabVanced, Bánki et al. conducted an online eye tracking study to assess 4- to 6-month-old infants' sensitivity to audio-visual synchrony. Braun et al. developed an app for the iPad that recorded videos of toddlers' responses to images corresponding to familiar and unfamiliar words. Children's looking time was later analyzed using a combination of human coding and neural networks. Eschman et al. described how existing deep learning tools for face recognition can be adapted to automatically code eye gaze from recorded sessions.

Other kinds of responses can be recorded in unmoderated sessions. Marimon et al. used LabVanced to collect reaction time from 3$\frac{1}{2}$ to 8-year-old children who responded with button presses to assess their sensitivity to non-adjacent dependencies in linguistic stimuli. Ross-Sheehy et al. used Gorilla to record button presses from 4- to 10-year-old children in a change detection task as a measure of their visual working memory. Chere and Kirkham investigated executive functions in contexts of noise with 11–18 year-olds on Gorilla, in which both accuracy and reaction time measures were collected.

Another approach to unmoderated research was to train caregivers to collect data in their homes or during their daily lives. Franchak et al. demonstrated how they could study infants' body positions using a set of wearable inertial sensors delivered to the infants' homes and applied by the parents and developing neural-network based analyses of these body postures. Van den Heuvel et al. discussed the value and pitfalls of experience sampling methods (ESM) using smartphones to gather data on infants and their families.

## Comparison of in-Person vs. Remote Data Collection

Regardless of the particular data collection procedure, an important question is how the results of data collected remotely compares to data collected in-person. Given the lack of control in the testing environment–and the presence of many more distractions than in the lab–it is not immediately obvious whether data collected remotely will yield the same results as data collected in person in a lab setting. This central question was explored by a large proportion of authors, and the results were mixed.

One issue is simply whether the quality of the data are comparable to those collected in the lab. It would not be surprising if data collected online were noisier, as there are many variables that are difficult to control (e.g., distractions, lighting, and quality of recording device). On the other hand, children may be more comfortable at home, and thus online data collection may actually have less noise.

For some procedures, the data quality for online studies was quite good. Bacon et al. reported that data loss in a looking-while-listening task was similar to that observed in the lab. Morini and Blair reported similar numbers of trials from preschoolers in the lab and tested online in a vocabulary learning task (using looking as a measure) with preschoolers. But others reported poor data quality from online sessions, for example when remotely conducting eye-tracking (Bánki et al.) or recording audio responses (Gijbels et al.). There are remedies to some sources of poor data quality, however. Gijbels et al. for example, provided children with wearable audio recorders (LENA, Xu et al., 2009) to obtain higher quality audio data than can be obtained from Zoom recordings.

A second issue is whether the same patterns of results are observed in both contexts. Several studies found no differences between data collected in remote and in-lab sessions. Attempts to replicate previously collected (and often published) findings from lab-based research were successful. For example, in a moderated task using Gorilla, Yamamoto et al. replicated previous findings from lab-based studies for children's emotion perception in auditory and visual stimuli. Vales et al. used a Qualtrics task in a moderated session and replicated previously reported findings about 4- to 6-year-old children's semantic knowledge. Schidelko et al. reported results from online false belief tasks with preschoolers that replicated previous findings. However, Bochynska and Dillon conducted a visual preference study with infants using Lookit, and did not replicate findings on infant shape discrimination from data collected in the lab.

Others directly compared data collected in the lab and online. In some cases the procedures and methods were very similar, as were the results. For example, Segura and Pompéia compared results when 9- to 15-year-old children were administered a battery of executive function tasks by an experimenter, either in person or moderated online, and observed no differences in performance. Morini and Blair found no differences in a looking task assessing vocabulary learning in toddlers when conducted online or in person. Silver et al. found that 2- to 3-year-old children responded similarly in a number tasks given online or in the lab. Chuey et al. replicated a number of studies of social cognition in young children using in-lab and remote testing methods. In other cases, the results differed in the two contexts. Not only did Bánki et al. find different quality of eye tracking recorded online and in person, they also obtained different patterns of results. In a comparison of performance on a second order inference task conducted in the lab, in a supervised online task, and in an unsupervised online context, Lapidow et al. observed that the online findings were weaker, and only oldest children tested show above chance performance in that context. In Bacon et al.'s looking-while-listening task (coded frame by frame through Zoom), both accuracy and reaction times showed differences from in-lab studies, with toddlers faster and more accurate in the Zoom study.

In summary, although some findings are robust to differences in testing context, others are not. This observation has implications both for how we think about specific findings–and whether or not they are robust and replicable–and also for what kinds of questions must be asked in a lab context and what kinds of questions can be asked utilizing remote methods.

## Online Data Collection Challenges and Best Practices

A significant contribution of the papers in this Research Topic is what was learned and how online remote testing can be effective, which we discuss in the following.

### Adapting Procedures for Online Testing

As many of us discovered early in the COVID-19 pandemic, setting up an online study is not necessarily easy or fast. Many online platforms, such as Lookit or LabVanced, require learning new paradigm construction tools. When using less technically demanding platforms, such as Zoom, researchers discovered the importance of testing internet speed (e.g., Bacon et al.; Eschman et al.) or the limitations of some aspects of the recording for obtaining high quality data (Gijbels et al.). The challenges are not just technical, however. Researchers must consider how their tasks and procedures must be adapted for administration remotely and online. For example, Krogh-Jespersen et al. described how they adapted the Mullens, which is a standardized tool that requires using specific materials. They used parents as test administrators and adapted materials for presentation using PowerPoint, eliminating items that could not be tested remotely.

Several of the papers in this Research Topic provide guidance for the decisions researchers need to make when considering moving their task or procedures online. Kominsky et al. provide guidance to decide on whether moderated or unmoderated procedures are best, for example considering the importance of experimenter involvement vs. the convenience of participants completing the study on their own schedule. Shields et al. provide an overview of some of the platforms available for online, unmoderated testing, which vary in their expense, the responses that can be recorded, and the ease with implementing new procedures. Braun et al. show the advantage of developing custom-build solutions, if one's research team has the technical skills.

A significant consideration is stimulus presentation. Presenting stimuli remotely is more complicated than in the lab. Some researchers send stimuli or materials home to families, and record children interacting with those materials during moderated sessions (Kominsky et al.; Silver et al.). It is more common for researchers to present stimuli over the internet during moderated or unmoderated sessions, using screen sharing, downloading stimuli onto participants' computers, or streaming on the web. The different methods have different pros and cons, including lags and dropped frames, slow internet speeds, and temporal differences. Kominsky et al. describe how researchers must balance the need for control over stimulus presentation and the quality of the stimulus presentation.

In addition, interacting with subjects online is different from in person. Experimental tasks may therefore need to be changed. Because children's attention may be more difficult to maintain during online sessions than in the lab, the recommendation is that tasks are kept short and that experimenters elicit regular responses from children (in moderated tasks) to monitor children's attention (Chuey et al.; Shields et al.).

### Security Considerations

Online data collection requires that researchers consider data security. Information technology policies on University campuses frequently change, and requirements for how data collected from individuals can be stored and transmitted varies from institution to institution and from country to country. Basic questions such as what data can be collected, who has access to it, and how it is stored can be a challenge. The US has different standards and concerns than Europe, which may make collecting data in both environments difficult (Zaadnoordijk et al.).

As a result, researchers must consider carefully the platforms they adopt to collect data with children and families. Chuey et al. provide pros and cons of several popular video conferencing platforms for the purposes of data collection. For example, Zoom has security features such as real-time encryption, the ability to require a passcode and enable waiting rooms (Gijbels et al.; McElwain et al.; Shin et al.). It can allow researchers to record sessions directly onto their local harddrives (Bacon et al.; Segal and Moulson; Venkatesh and DeJesus), or to have participants record their sessions on their own hard drives (to avoid lags; Morini and Blair). In this second case, the researcher has to have a way to securely transfer the recording from the participant's computer to the researcher's computer. Regardless of how the research team solves these issues, online testing raises privacy issues as it often involves creating recordings that show parts of the participants' homes. That is, although online data collection can provide insight into children's environment (Chere and Kirkham) and how children behave while at home (Pochinki et al.), it also exposes the researcher to a new level of privacy and security concerns.

### Involving Caregivers in the Study Process

When testing participants online, the opportunities for instructions are more limited than in the lab, even in moderated sessions. In the absence of an experimenter and a lab setting, parents and other caregivers often play an important role in order to ensure adequate study setting and control. Shields et al. provide suggestions for how to involve parents in this way, and researchers in this Research Topic often emphasize the role of parents as active co-researchers (e.g., Eschman et al.; Zaadnoordijk et al.). How this could best be achieved depends on the required caregiver contributions and the task format; for instance, Krogh-Jespersen et al. emphasize the importance of creating rapport between caregivers and researchers in longer, moderated tasks, while shorter, unmoderated experimental protocols might especially benefit from clear instructions (Shin et al.).

For the latter, checklists and tutorial videos are recommended to ensure parents set up their home study environment in a way that minimizes interruption and distraction (Shin et al.). Another technique that researchers put forward is to do pre-study sessions with parents, including technical and equipment tests to check that parents use the correct devices and that quality of stimuli and internet speed were sufficient (Eschman et al.; Morini and Blair). What each of these examples illustrate is that involving and training the parent can have a positive impact on data quality and the overall success of remote data collection.

## The Promises of Remote Testing

The promise of remote data collection is enticing. Developmental scientists have long struggled with collecting ample sample sizes, as well as having samples that are diverse and representative of all children. In addition, remote data collection is more accessible to researchers who have limited space and resources to collect in-person data. Thus, although the COVID-19 pandemic motivated many to collect data online out of necessity, it is likely that many researchers will continue to collect data remotely even after it is possible to collect data in person.

The shift to online testing made it possible for developmental scientists to ask and answer questions that are difficult or impossible to address in-person in a lab. Remote research provides insights into children's lives at home that is only possible with remote testing. Pochinki et al., for example, showed how with remote testing we gain understanding into the kinds of puzzles preschoolers play with their parents, and the kinds of behaviors mothers and preschoolers engage in during that play. Chere and Kirkham assessed the impact of noisy home environments on executive functioning in adolescents, illustrating how remote testing can tap into aspects that are hard to assess in the lab. Franchak et al. collected extensive data about motor behavior during naturalistic interactions at home by sending home equipment and instructing parents how to use it at home. These papers illustrate how remote testing gives us insight into development in context in a way that lab-based research cannot.

Online methods also have promise for developmental screening, which is expensive for health services to conduct in-person. Giraldo-Huertas and Schafer compared a standardized developmental screening with a parental measure that could in principle be administered online. Nelson et al. directly compared how pre- and full-term children performed on standardized and experimental cognitive assessments at 4 and 5 years of age in person and online. They found no differences as a function of format on 5 of 8 tasks and found that there were no effects of format for children at risk.

One still at least partly unfulfilled promise of online data collection is a more global reach and inclusivity. For instance, Lookit, the main platform for infant looking time studies, is primarily available for families living in English language environments and under US data protection laws. Nevertheless, we think that this problem is more surmountable in online than in-lab settings, and indeed, projects like ManyBabies-AtHome (Zaadnoordijk et al.) aim to globally broaden access to relevant software and data management options. A related problem is recruitment, where again research recruiting English-speaking and US-based families can profit from quickly evolving platforms such as ChildrenHelpingScience, with equivalents for other areas only sparsely available (but see Kinder Schaffen Wissen for German speakers). Kato et al. tackle the problem of creating a database for recruiting infants and storing data online in Japan, including the creation of a researcher consortium to manage such efforts. Another concern for inclusivity in online studies is the necessity of a stable internet connection and a device to participate in studies. A lot of work still needs to be done to overcome these problems, but this Research Topic assembles some suggestions for solutions, such as lending participants a Wifi tool or hotspot or refer them to public places that offer free internet, or to create tasks that allow participants to participate over their mobile phone as opposed to a webcam-enabled computer (Shin et al.). Thus, while remote data collection is still not as global and inclusive as we might have imagined at the outset of the pandemic, the research community suggests and has started implementing concrete and attainable solutions toward this goal.

Even if researchers will solve the practical problems of testing a diverse subject population, online testing does not guarantee that diverse samples will be automatically recruited. For example Bacon et al. deliberately tested the idea that they could recruit a more diverse sample online by using microtargeting Facebook ads. However, this study also illustrates that although in principle online testing provides access to populations who would not ordinarily come to the lab (e.g., they live too distant), it takes effort and care to recruit more diverse populations, just as it would to recruit those samples for in-person testing. Liu et al. demonstrate the effectiveness of community engaged labs for recruiting diverse samples.

## Conclusion

Research at a distance is here to stay for developmental science. The collection of papers in this Research Topic illustrate many of the ways that methods and procedures can be adapted for remote administration. The papers provide models for solutions to common problems, and will help researchers in the future make decisions about how to conduct empirical research at a distance to answer key questions in developmental science.

## Author Contributions

ST and LO wrote the manuscript. All authors made substantial, direct, and intellectual contributions to this work and approved it for publication.

## Funding

This work was supported by an ERC Advanced Grant ERC-2017-ADG, FOUNDCOG, 787981 to RC, as well as a JSPS Grant-in-aid for Specially Promoted Research (20H05617), JSPS Grant-in-aid for Transformative Research Areas (20H05919), and a JST-ActX grant in the research area AI powered Research Innovation/Creation awarded to ST.

## Conflict of Interest

The authors declare that the research was conducted in the absence of any commercial or financial relationships that could be construed as a potential conflict of interest.

## Publisher's Note

All claims expressed in this article are solely those of the authors and do not necessarily represent those of their affiliated organizations, or those of the publisher, the editors and the reviewers. Any product that may be evaluated in this article, or claim that may be made by its manufacturer, is not guaranteed or endorsed by the publisher.
